# Guided vascularization in the rat heart leads to transient vessel patterning

**DOI:** 10.1063/1.5122804

**Published:** 2020-03-05

**Authors:** Eileen L. Brady, Mitchell A. Kirby, Emily Olszewski, Parker Grosjean, Fredrik Johansson, Jennifer Davis, Ruikang K. Wang, Kelly R. Stevens

**Affiliations:** 1 Department of Bioengineering, University of Washington, Seattle, Washington 98195, USA; 2 Institute for Stem Cell and Regenerative Medicine, Seattle, Washington 98195, USA; 3 Department of Pathology, University of Washington, Seattle, Washington 98195, USA; 4 Department of Ophthalmology, University of Washington, Seattle, Washington 98104, USA

## Abstract

Recent progress in the production and maturation of iPSC-cardiomyocytes has facilitated major advances in building bioartificial heart tissue with functional cardiomyocytes. Despite this progress, vascularizing these constructs continues to be a barrier to clinical application. One emerging strategy for vascularization uses aligned “cords” of endothelial cells in tissue grafts to guide assembly of chimeric microvessels upon graft implantation. Here, we test whether this approach can guide vascularization of a bioartificial tissue implanted on the rat heart. We find that patterned cords of human endothelial cells anastomose and become perfused with host blood by 3 days post-implantation. Immunohistochemical staining confirmed that graft-derived micro-vessels persist in the patch for 7 days. Furthermore, we noted a shift in distribution of vessels in the patch from patterned cord-associated clustering at 3 days to a more diffuse distribution pattern at 7 days. This loss of patterning corresponded to an infiltration of CD68+ cells and an increase in collagen within the patch. Upon further engraftment of patches containing both cords and human cardiomyocytes, we identified human cardiomyocytes and graft derived vasculature at the time of explant. Our findings show that patterned endothelial cords guide transient vessel patterning on the rat heart. Our results also suggest that future work should be directed at further adapting vascularization strategies to the epicardial environment and add to an important emerging dialog in cardiac cell therapy that points to the need to characterize host response prior to or in parallel with efficacy studies.

## INTRODUCTION

The only definitive treatment for end stage heart failure is replacing the damaged muscle via heart transplant. However, the supply of transplantable organs is limited in comparison to the number of patients who could benefit from transplant.^1^ For patients who do not have access to a deceased donor heart, bioartificial cardiac tissues offer a promising alternative source of implantable human myocardium. Recent advances in scalable production and maturation of pluripotent stem cell derived cardiomyocytes have brought this technology closer than ever to clinical translation.^2–7^ A remaining obstacle is the inability to generate a dense, host-perfused vasculature that can support oxygen and nutrient exchange for implanted cardiomyocytes,^8^ without which the tissue thickness is limited by the diffusion limit of oxygen, preventing the creation of human-scale tissues for clinical applications.

Early success toward vascularization by Stevens *et al.*^9^ and Levenberg *et al.*^10^ was achieved through homogenously seeding endothelial and supportive stromal cells into engineered tissues, which led to self-assembled tubular networks that became perfused upon implantation.^9,10^ However, some subsequent studies have suggested that the disorganized and tortuous nature of self-assembled networks may make them susceptible to thrombosis.^11^ Therefore, we and others have worked to develop methods to more precisely control geometry of engineered vascular networks.^12–14^ One such method is micropatterning endothelial cells and stromal cells along with collagen to form aligned cord-like structures.^12^ We showed that such endothelial cords improved vascularization and function of bioartificial liver tissues implanted in the mouse abdomen by guiding host vessel ingrowth and decreasing time to perfusion.^15^ This method of vascular patterning could be appealing in the context of the injured heart, as the cords may be able to recruit vasculature from the peripheral healthy tissue into the hypovascular infarct zone. However, it is not known whether cords can guide vascularization in the environment of the supra-epicardial surface, where an engineered tissue patch would be applied. Therefore, we sought to determine if cords could guide vascular infiltration of a bioartificial tissue patch applied to the surface of the rat heart.

Here, we demonstrate that endothelial cords embedded within a fibrin hydrogel induce formation of aligned micro-vessels supra-epicardially on the rat heart. We found early anastomosis and patterning of nascent human derived blood vessels within these tissues. However, we also observed a robust host response and loss of architectural vascular patterning over time. Thus, this work suggests that guided vascularization may be a useful strategy for cardiac tissue engineering and informs future directions in adapting vascularization strategies for grafting on the epicardium.

## RESULTS

### Culture of endothelial and stromal cells in microchannels facilitates formation of cords

**FIG. 1.**
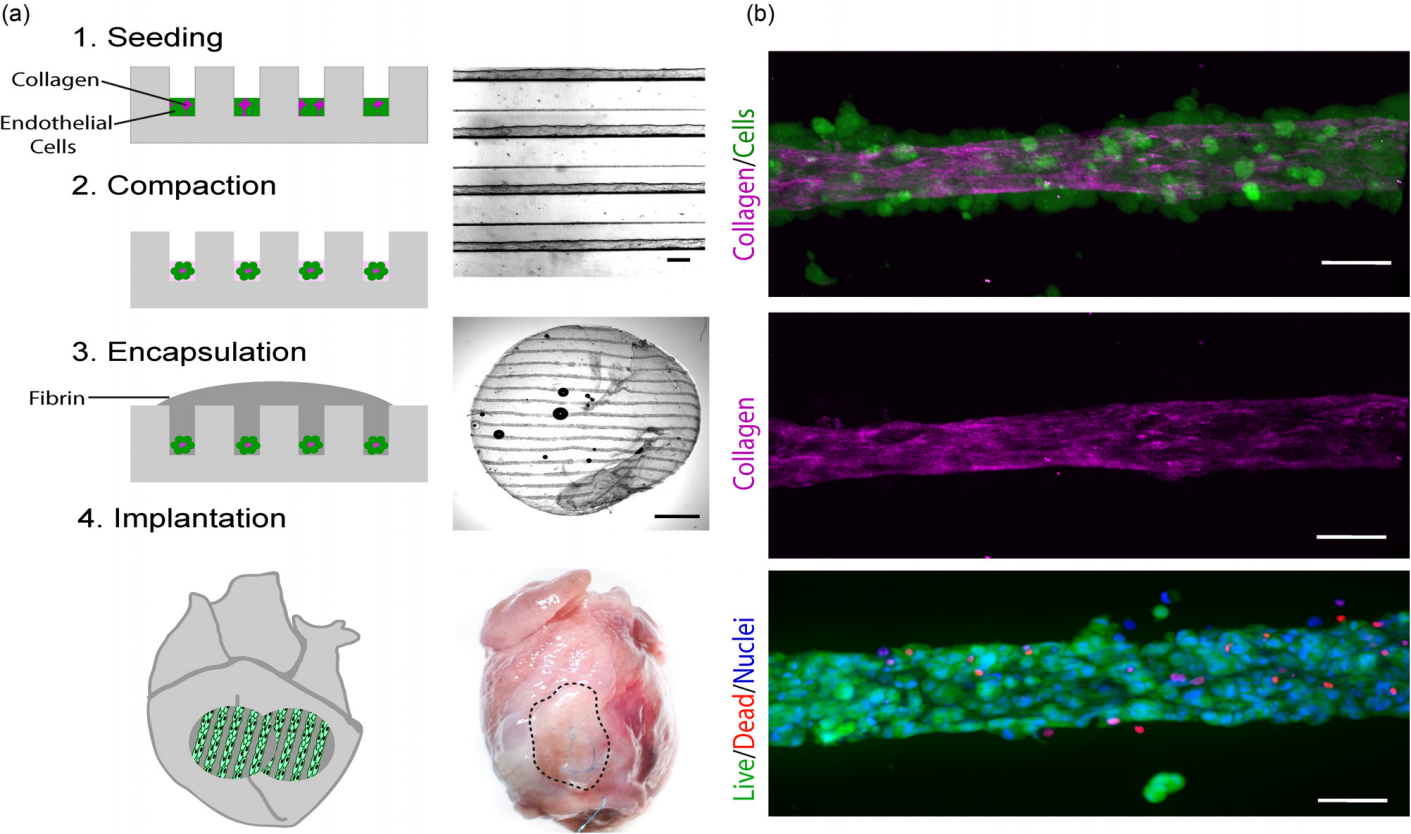
Generation of patches containing “endothelial cords” for epicardial implantation. (a) Fabrication of patches by molding of endothelial cords, encapsulation in fibrin, and implantation on the supra-epicardial surface of an athymic rat heart. Scale bar (top) = 50 *μ*m. Scale bar (middle) = 1 mm. (b) *In vitro* characterization of cords with staining of live (green; calcein) and dead (red; ethidium homodimer) cells and second-harmonic generation microscopy to visualize collagen (pink). Scale bar = 50 *μ*m.

We first cultured human umbilical vein endothelial cells (HUVECs) and a stromal cell population (CH310t1/2) within 2.5% collagen in PDMS microchannels with a width of 150 *μ*m and a height of 100 *μ*m as published previously.^12^ We found that after a 3-h incubation within the channel molds, cells compacted to form aligned cords with approximately half the diameter of the original channels [Fig. 1(a)]. Second harmonic generation microscopy^16^ demonstrated that compacted cords consisted of a loosely consolidated collagen core, with cells adhered around the periphery [Fig. 1(b)]. We encapsulated cords within a fibrin hydrogel to generate implantable bioartificial tissues and used a 6 mm biopsy punch to generate patches for implantation [Fig. 1(a)].^13^ We confirmed cellular viability of encapsulated cords by staining for live and dead cells at the time of implantation [Fig. 1(b), bottom].

### Cords induce guided vascularization on the uninjured rat heart

**FIG. 2.**
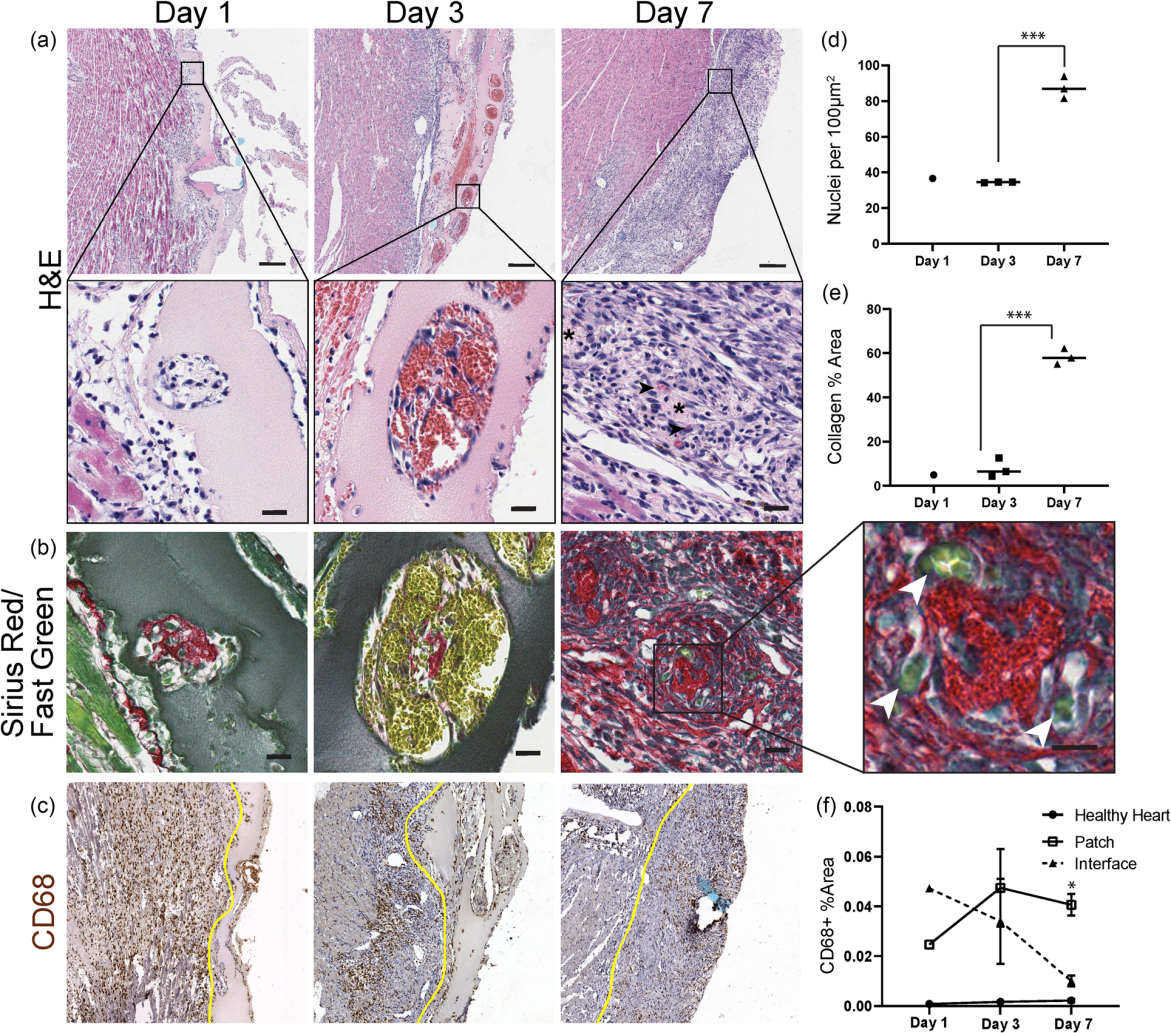
Cord patches become perfused at 3 days post-implantation. (a) H&E and (b) Sirius Red/Fast Green staining of cord patches explanted at 1, 3, and 7 days indicates pooling of blood around cords at day 3, followed by remodeling into micro-vessels (arrowheads, day 7). Scale bar = 20 *μ*m, and inset scale bar = 10 *μ*m. (c) CD68+ cells are prevalent at all timepoints, remaining elevated in the patch at 7 days. The yellow line indicates the patch boundary. Scale bar = 100 *μ*m. (d) Hematoxylin positive nuclei within the patch per 100 *μ*m^2^. (^***^p < 0.001). (e) Quantification of the collagen area from Sirius Red stain. (^***^p < 0.001). (f) Quantification of the CD68+ area (^*^p < 0.05). Error bars indicate S.E.M., n = 3 (day 3, day 7), and n = 1 (day 1).

We next sought to determine if bioartificial tissues containing micromolded endothelial cords could become vascularized on the heart, as we found previously when similar constructs were implanted in the abdomen of mice.^13,15^ To characterize vascularization of cord-containing tissue, we implanted patches supra-epicardially in athymic rats and harvested the hearts at 1, 3, 7, and 14 days post implantation. After 7 days and 14 days of implantation, we noted fibrous adhesions between the heart and the chest wall, which we carefully dissected away to free the heart for explant. Patches were located by visualizing the suture knot on the exterior of the heart through seven days of engraftment. While we were able to locate the implanted patches by identifying suture at 14 days, no patch was evident by gross examination, and so we did not further analyze this timepoint. Using routine histological analysis of patch sections with Hematoxylin and Eosin (H&E), we clearly identified a linear array of cords' cross sections that were surrounded by pools of blood at 3 days [Fig. 2(a), center panels]. Upon examination of individual cords, we found numerous, large blood-filled lumens arranged circumferentially around a central point. The blood pools were not apparent at the 7 day timepoint. Instead, we noticed a subset of cords associated with small, <10 *μ*m microvessels [Fig. 2(a)]. The cord cross sections at 7 days had a characteristic eosinophilic, acellular core with micro-vessels situated peripherally around this core. We also confirmed identification of cord-associated micro-vessel formation with Sirius Red/Fast Green staining, in which micro-vessels were visualized as tight consolidations of blood (yellow) at the periphery of a collagen core (red) [Fig. 2(b), inset]. We and others have shown that cord-associated blood pools mature to form stable microvessels in tissues implanted in the mouse abdomen^13^ (Fig. S1). Therefore, our results suggest that a similar process of guided vascularization can occur on the heart.

In addition to revealing early infiltration of blood associated with cords, H&E staining showed a significant increase in the density of hematoxylin positive nuclei within the patch between 3 and 7 days, suggestive of an inflammatory host response [Fig. 2(d)]. Correspondingly, the lightly eosin-positive fibrin identifiable at day 1 and day 3 appeared to be largely absent by day 7 [Fig. 2(a)]. To further characterize the apparent immune response, we immunostained for CD68, a macrophage marker. At day 1, we found marked infiltration of CD68+ cells at the patch periphery and extending several hundred micrometers into the heart tissue underlying the patch [Fig. 2(c)]. At the day 3 and day 7 timepoints, we continued to observe CD68+ cells at the patch-heart interface and increased infiltration of CD68+ cells within patch boundaries. To better understand these trends, we quantified the CD68+ area within the patch, at the heart-patch interface, and in tissue from a presumably healthy unperturbed area of the heart [Fig. 2(f)]. As expected, only rare CD68+ cells were observed within the distant healthy heart at all timepoints. Within the patch-heart interface, defined as 300 *μ*m underlying the patch, the CD68+ area was initially elevated but was not significantly higher than healthy heart at 7 days. In contrast, the CD68+ area within the patch remained elevated at levels significantly higher than healthy heart at 7 days.

To characterize the composition of the patch at 7 days, we stained for collagen with Sirius Red and counterstained with Fast Green [Fig. 2(b)]. At day 1 and day 3, we identified collagen at the center of the cords, consistent with our *in vitro* results. However, by day 7, collagen was identified both in the cords and throughout the patch, and the percent of patch area positive for Sirius Red had increased [Fig. 2(e)]. These results suggest that by day 7, the original fibrin scaffold had been degraded and replaced at least in part by a dense cellular milieu and collagen, likely largely derived from the host.

**FIG. 3.**
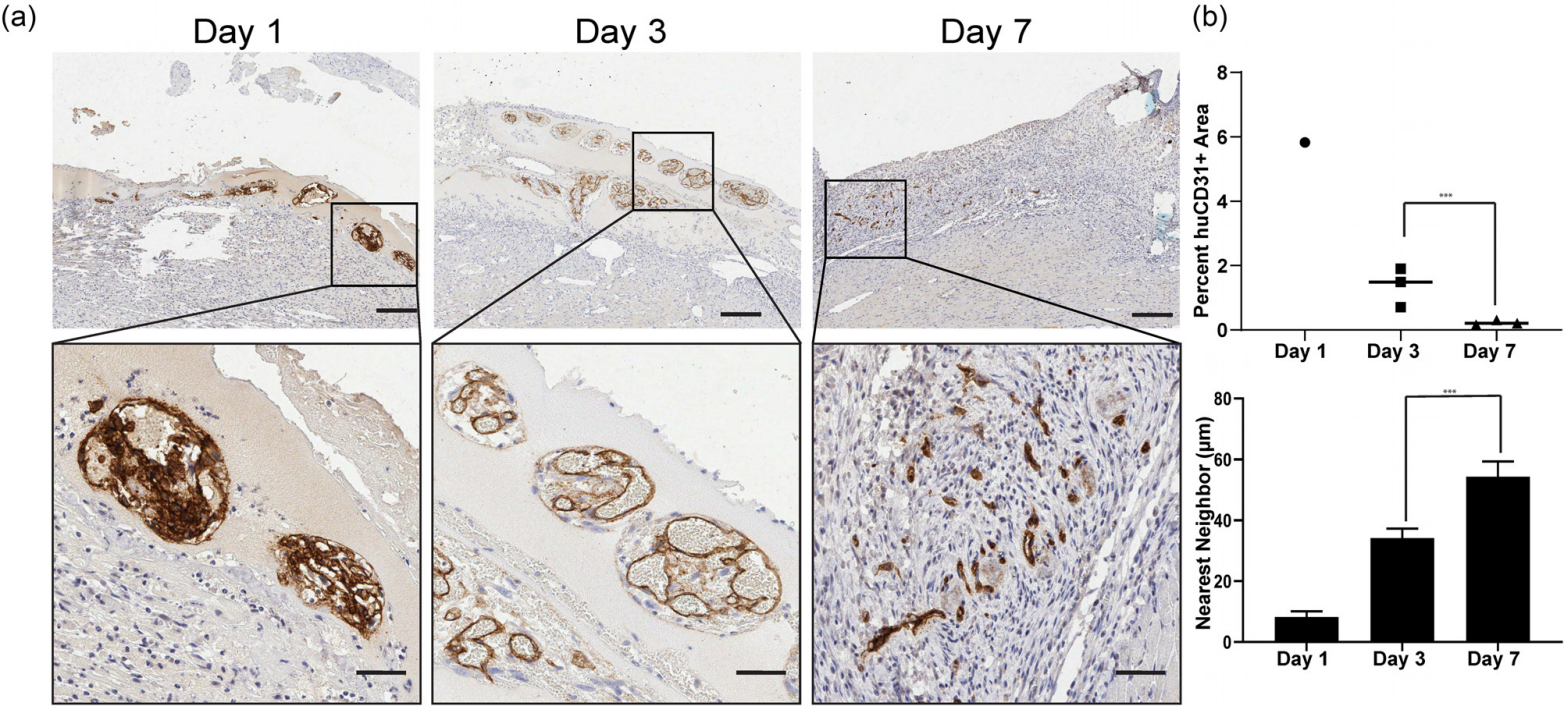
Human CD31+ cells form large lumens that reorganize into micro-vessels. (a) huCD31 staining of explants from 1, 3, and 7 days. Scale bar (top) = 200 *μ*m. Scale bar (bottom) = 50 *μ*m. (b) Quantification of the huCD31 staining area as the percent of patch over time (top). Nearest neighbor analysis of huCD31 staining at 1, 3, and 7 days (bottom). (^***^p < 0.001). n = 1 for day 1 and n = 3 for day 3 and day 7. Error bars indicate S.E.M.

Next, we wanted to determine if the structures associated with the cords contained graft derived endothelial cells. Immunostaining for human CD31 identified human endothelium at 1, 3, and 7 days [Fig. 3(a)]. At day 1, the huCD31+ stained area was densely packed in cross sections of cords. By day 3, the staining indicated an expansion of the human endothelium to form large lumens, similar to previous observations of blood-filled lumen formation in mouse abdominal cords implants.^13^ The area of huCD31+ cells as a percentage of patch area had decreased by 7 days, but the remaining cells had assembled as the interior lining of small lumens consistent with mature micro-vessel formation [Fig. 3(b)]. We noted the huCD31+ structures were no longer tightly associated with cords, as had been seen at 3 days. Instead, we found huCD31+ micro-vessels more diffusely arranged in the patch [Fig. 3(b), bottom].

### Cords support formation of blood filled huCD31+ lumens

**FIG. 4.**
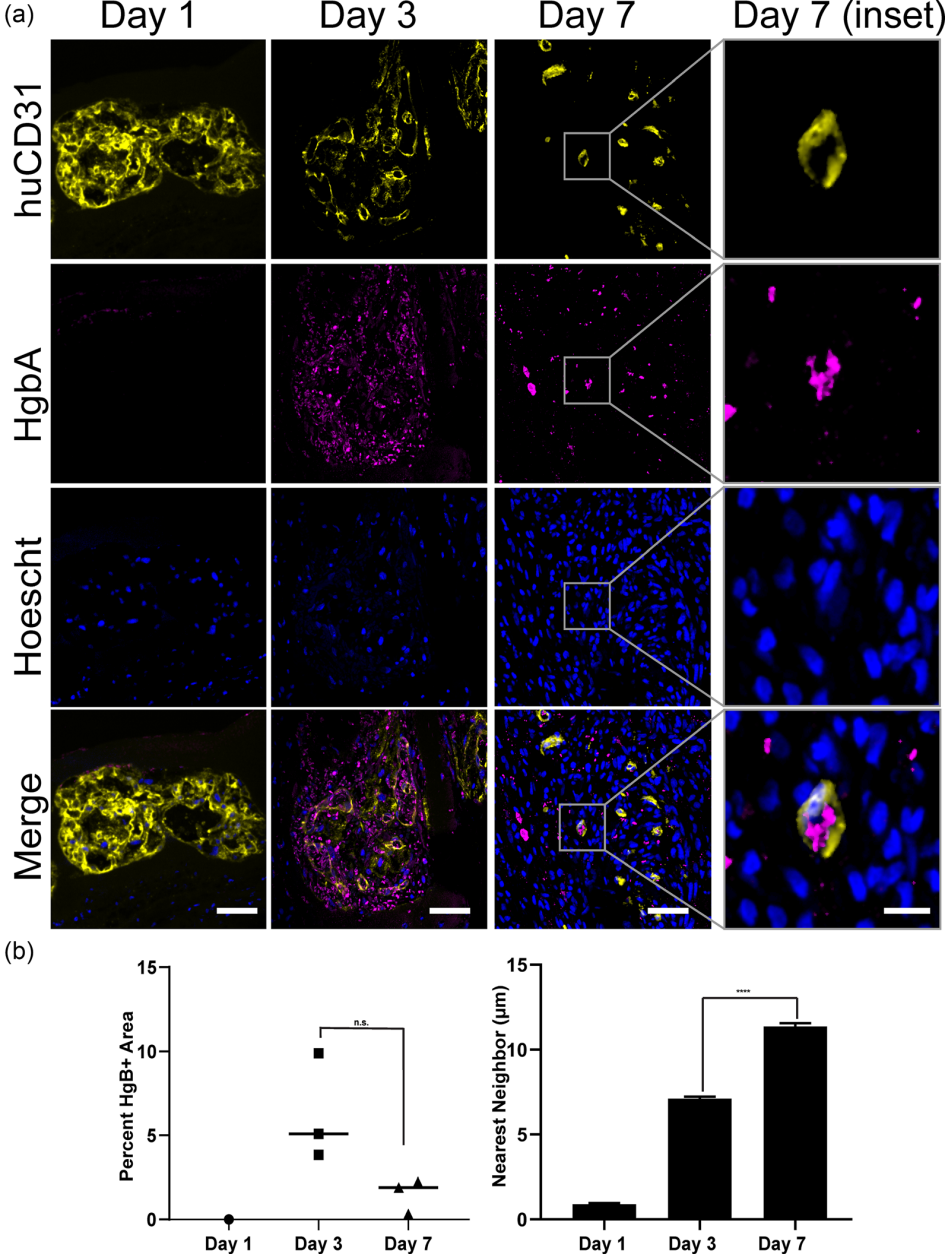
Graft-derived vessels are perfused with blood. (a) Co-staining for HgbA and huCD31 in explants from 1, 3, and 7 days. Scale bar = 50 *μ*m. Inset scale bar = 10 *μ*m. (b) Quantification of HgbA staining as the percent of patch area and distance to the nearest neighbor. (^***^p < 0.001). n = 1 for day 1 and n = 3 for day 3 and day 7. Error bars indicate S.E.M.

H&E and Sirius Red staining suggested the presence of blood-containing lumens associated with cords. Thus, we next sought to confirm the erythroid identity of these cells. Immunostaining for Hemoglobin A (marks rat red blood cells) confirmed the presence of blood within the graft at day 3 and day 7, but not at day 1 [Fig. 4(a)]. Co-staining for both huCD31 and HgbA identified lumens lined with human endothelial cells that contained rat blood at day 3 and day 7. At day 3, we noted ample blood present both within and proximal to the large huCD31+ lumens [Fig. 4(a)]. By day 7, we again found smaller huCD31+ vessels, some of which contained blood [Fig. 4(a)]. While there was not a significant change in the total blood area within the patch between day 3 and day 7, we noticed a shift in distribution of both HgbA and huCD31 staining from highly clustered at 3 days to more disordered by 7 days [Fig. 4(b)]. To quantify this observation, we performed nearest-neighbor analysis of the huCD31 [Fig. 3(b)] and HgbA stains [Fig. 4(b)]. This analysis confirmed a decrease in clustering of these markers between 3 and 7 days.

#### Co-engraftment of human myocardium and microvessels on the rat heart

**FIG. 5.**
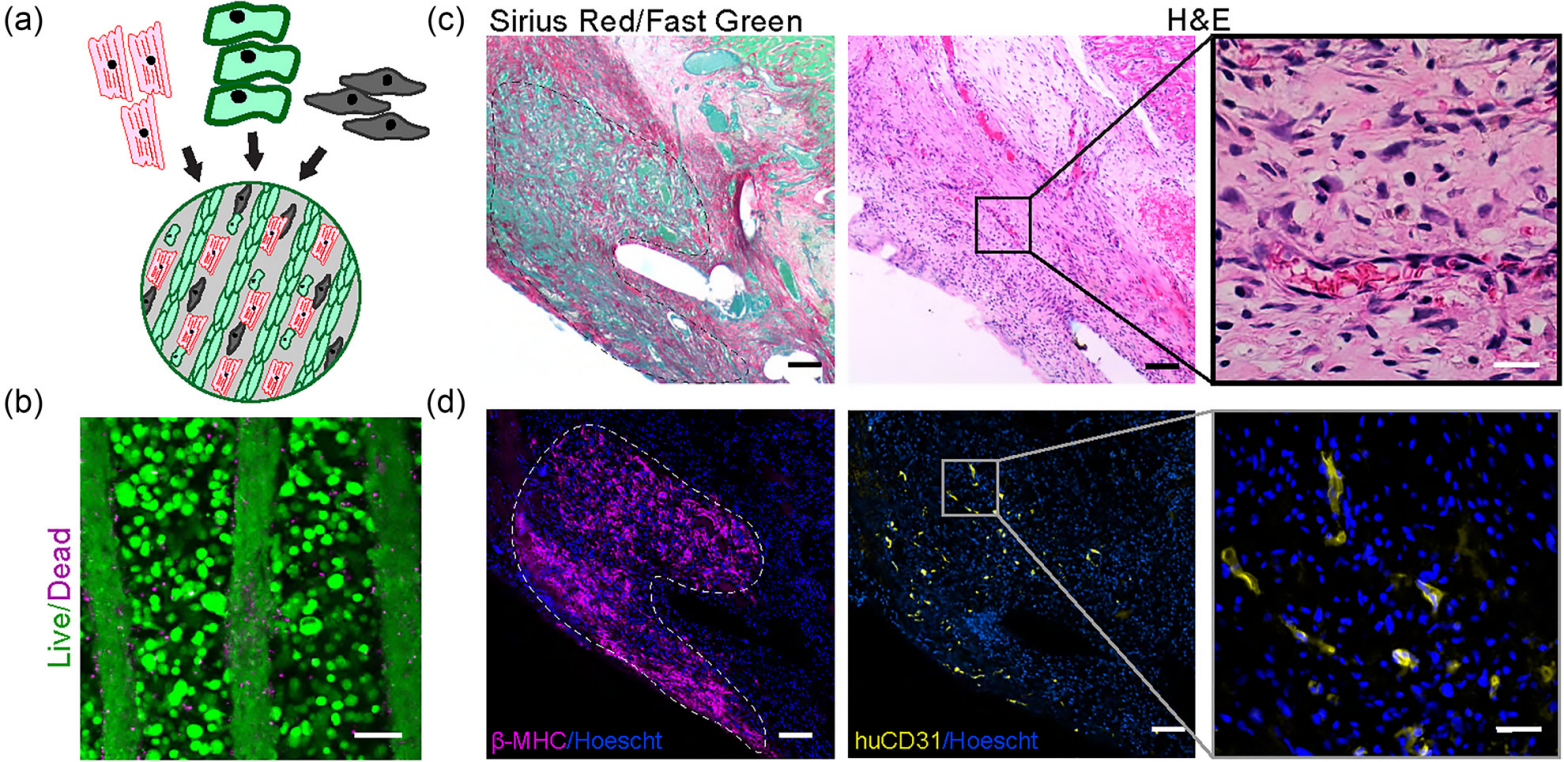
Cord-containing cardiac patch engrafts on rat heart. (a) Schematic showing the composition of the cardiac patch and (b) live/dead staining of the patch at implantation; scale bar = 50 *μ*m. (c) Sirius Red staining of the patch at day 10 (left) and H&E staining of the patch at day 10; the inset shows microvessels (right). (d) Immunostaining of human graft (β-MHC) and endothelial cells (huCD31). Scale bar = 50 *μ*m. Scale bar (inset) = 10 *μ*m.

As our ultimate goal is to induce vascularization of human engineered cardiac tissues, we sought to test whether cords could be integrated within a multicellular human myocardial patch. We adapted the basic cord patch fabrication protocol for this purpose by including human iPSC-cardiomyocytes, stromal cells (Normal Human Dermal Fibroblasts, NHDFs), and endothelial cells (HUVECs) within the fibrin bulk surrounding the patterned cords [Fig. 5(a)]. Staining of viable and non-viable cells within the cardio-cords patch confirmed that cells within the cords, and in the fibrin bulk, were viable after fabrication [Fig. 5(b)]. We then implanted patches on the surface of athymic rat hearts. Similar to the 7 day timepoint in previous experiments, we identified a collagen rich patch overlying the implant site [Fig. 5(c)]. As we had found in our previous studies, H&E staining confirmed the presence of micro-vessels throughout the patch area [Fig. 5(c), right]. However, unlike the grafts containing only cords, the Sirius Red/Fast Green staining of cardio-cord grafts revealed a heterogenous staining pattern [Fig. 5(c), left]. Against a background of dense Sirius-Red positive collagen, we identified several relatively large collagen-deficient, fast green positive islands. When we stained these sections for human myocardium using the marker β-myosin heavy chain, we were able to correlate these collagen-deficient islands with areas of engrafted cardiomyocytes [Fig. 5(d), left]. We also identified human CD31 positive vessels, indicating a graft contribution to the vasculature [Fig. 5(d), right].

**FIG. 6.**
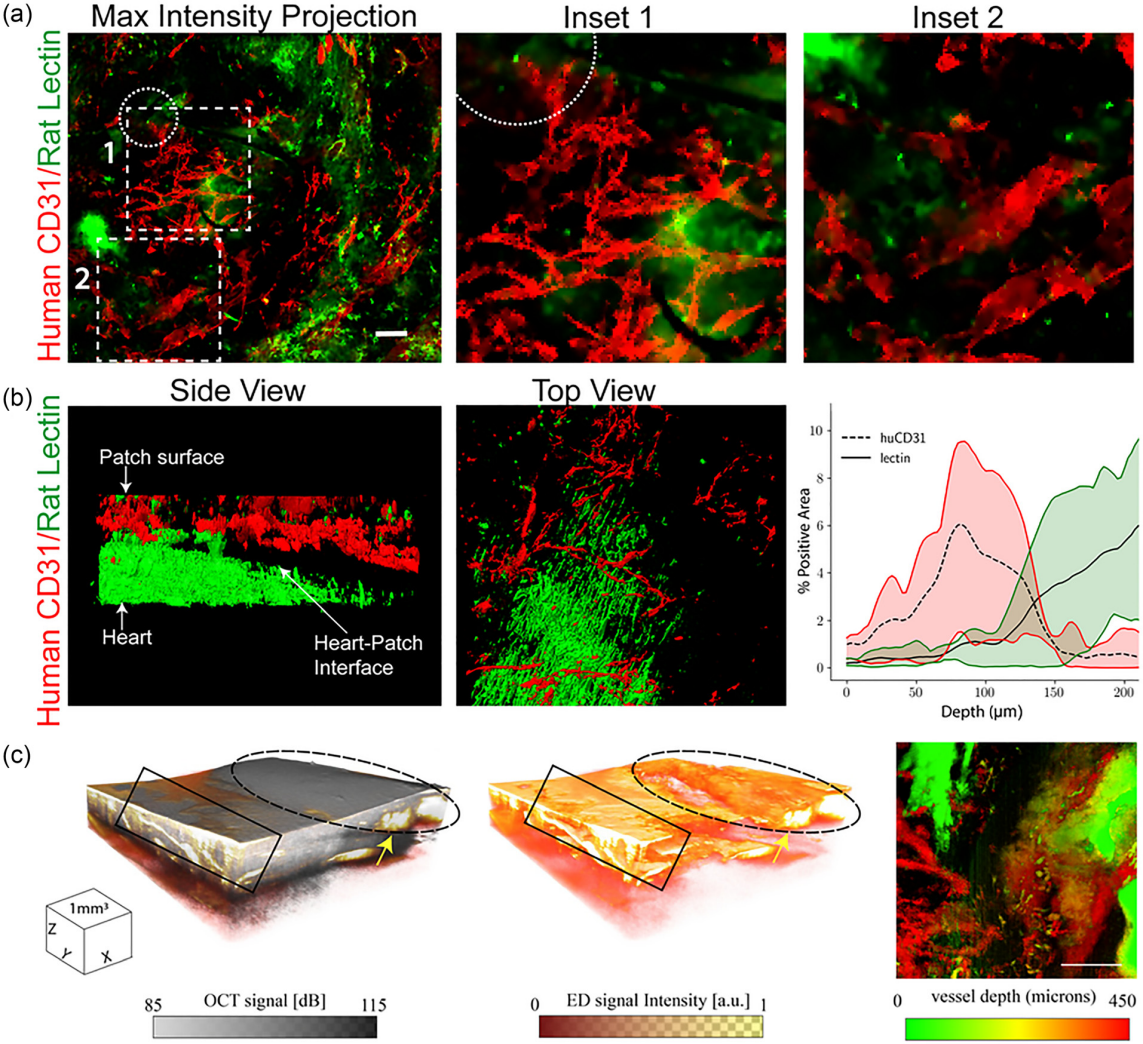
(a) Maximum intensity projection of 200 *μ*m z-stack of rat vessels stained with rodent specific lectin (LEL, green) and human vessels stained with an antibody for human CD31 (red). The dotted circle indicates the location of suture. Scale bar = 100 *μ*m. (b) 3D visualization of segmented data from rodent specific lectin (green) and human CD31 (red) staining. Quantification of the area positively stained for human (huCD31) or rat (lectin) vessels in the patch and adjacent heart, range indicated by the shaded region n = 3. (c) 3D cube of OCT structural information overlaid with the eigen decomposition (ED)-based OMAG signal showing flow through a network of irregular micro-vessels (arrow) in the patch region (dashed ellipse). 3D cube of the ED-based OMAG signal showing only flow (middle panel). Intralipid leakage (box) at the patch boundary can clearly be seen in the OMAG. En-face OMAG maximum intensity projection (MIP) image (right) of the vasculature (scale bar = 1 mm). Deeper vessels (red) can be seen connected to surface vasculature.

Although traditional tissue sectioning and histological imaging allowed us to identify human endothelial cells in patch sections, these methods did not provide information as to whether these cells reside within interconnected vascular networks that are patent and perfusable. To first better understand the 3D structure of patch derived vasculature, as well as the contribution of human vs rat vessels after 7 days of implantation, we turned to emerging 3D tissue “clearing” and visualization methods. First, we Langendorff perfused hearts with rodent specific (LEL) lectins. Next, we immunostained the intact hearts with a human-specific CD31 antibody. Samples were cleared with clearing-enhanced 3D microscopy (Ce3D)^17^ and imaged to obtain a 200 *μ*m thick z-stack through the patch [Fig. 6(a)]. We identified interconnected networks of human derived endothelial cells without obvious cord patterning, as well as sparse rodent vessels, within the patches at 7 days post implantation [Fig. 6(a)]. To improve the signal to noise ratio, images were segmented into stained and unstained regions using the machine learning based image analysis tool Ilastik.^18^ Processed data revealed the boundary between the patch and healthy heart to be approximately 150 *μ*m from the patch surface [Fig. 6(b)]. Quantification of human and rat contributions to the vessel network revealed primarily huCD31+ vessels within the first 100 *μ*m from the patch surface [Fig. 6(b)]. A transition zone with both human and rat contributions was evident from approximately 100 *μ*m to 150 *μ*m from the surface, while rodent vessels from the rat heart predominated deeper than 150 *μ*m. Therefore, vessels within the patch itself appeared to be primarily human derived, with some rat contribution deeper in the patch.

Next, we sought to determine whether the vessels within the patch were patent and perfusable. To do this, we used the optical coherence tomography (OCT)-based technique of optical microangiography (OMAG),^19^ which provides information on both the microvascular tissue structure and velocimetry. Imaging of patches at 7 days post implantation confirmed flow in the patch with a network of irregular microvessels near the surface of the tissue [Fig. 6(c)]. We also noted some regions of leakiness at the patch-heart interface. Together, these results demonstrate that patches contain interconnected networks of human endothelial cells and contain perfusable vessels at 7 days post implantation.

## DISCUSSION

Inefficient vascularization of engineered tissues remains a barrier to clinical translation. While early studies demonstrated that randomly seeded endothelial cells within engineered tissues can form rudimentary vascular networks, these networks are often transient and prone to failure.^11,20^ Multiple studies have now shown that geometric patterning of vascular networks in engineered tissues leads to improved vascularization.^13,14,20^ In this study, we have demonstrated that a bio-artificial tissue containing pre-formed cords of endothelial cells forms graft-derived microvessels containing blood when implanted on the supra-epicardial surface of the rat heart. At day 3, we noted robust patterning of nascent vessels. The large irregular lumens of these human CD31+ neovessels were filled with blood and appeared in tight association with patterned cords, which we identified by their Sirius-Red positive collagen cores. This initial process of cord-driven vascularization thus appeared morphologically similar to the process reported in mouse abdominal cord implants.^13^

In previous studies of endothelial cell (EC) cords, these initial large lumens remodel to form an array of host-graft chimeric micro-vessels tightly clustered around the cords by 7 days post implantation. Interestingly, although we did find capillary to arteriole sized microvessels throughout our patch at 7 days, these vessels were not closely associated with cords. These results suggest that in the context of the rat heart, cords induce transient patterned vessel formation at 3 days, rather than persistent patterning as has been reported in the mouse abdomen.^13^ Although we did not observe persistent patterning, we confirmed that vessels within the patch were perfusable at 7 days post-implantation.

This progressive loss of patterning corresponded to what appeared to be a robust inflammatory response. Interestingly, others have noted a heightened inflammatory response to supra-epicardial implants compared to implants in other locations.^21,22^ Studies using other methods to pattern vascular networks have also observed loss of vascular patterning geometry after implantation on the heart.^14^ Together, these data lead us to suggest that increased inflammation in response to implantation on the heart may cause patch degradation, collagen deposition, and loss of patterning. Despite this change in the patch composition, we found large dense human cardiac grafts in patches containing endothelial cords, suggesting that the inflammatory environment still allowed engraftment. In addition, the areas of engrafted cardiomyocytes had notably less collagen deposition than surrounding areas devoid of cardiomyocytes. We note that the experiments here differ from previously published work on cord-containing tissues not only in the implant location (abdominal vs supra-epicardial) but also in host species (mouse vs rat). Therefore, although we suspect increased inflammation associated with the cardiac implant site is driving the observed differences, species-specific effects could provide an additive or alternative explanation. Future studies will need to determine whether vascular patterning is functionally advantageous in the context of the heart, compared to unorganized vascular networks. Perhaps, more interesting will be investigation of the role of macrophages in vascular patterning and cardiac repair. As the recent work of the Molkentin laboratory indicates, an acute macrophage predominant response to cellular therapies in the context of the heart may in fact be critical for the success of these therapies.^23^

Together, our studies demonstrate the ability to transiently guide vascular patterning within bioartificial tissues engrafted on rat hearts. We speculate that inflammation leads to loss of scaffold architecture and rapid remodeling of the engineered construct in the heart compared to other implant locations. Concerningly, the problem of inflammation is likely to be exacerbated for injured hearts, where a wound healing response is already active. Thus, further characterization and ultimately attenuation of host response may be critical for the field of cardiac tissue engineering.^24^

## METHODS

### Cell culture and fabrication of cord patches

Human umbilical vein endothelial cells (Lonza, P2–P4) were cultured in EGM-2 media (Lonza) on tissue culture treated poly(styrene). Mouse mesenchymal stem cells CH310t1/2 (ATCC, P4–P7) were cultured in low glucose Dulbecco's Modified Eagle's Medium (DMEM) supplemented with 10% v/v fetal bovine serum (FBS, BioWest). Media was replaced every 48 h, and cells were passaged or used for experiments at 80% confluence.

Patches containing endothelial cords were fabricated as previously described.^12,13^ Briefly, a PDMS mold patterned with a parallel array of 150 *μ*m channels was treated for 5 minutes with 0.02% Pluronic F-127 and subsequently rinsed with phosphate-buffered saline (PBS). HUVECs and CH310t1/2 cells were resuspended at a 50:1 ratio in liquid 2.5% collagen neutralized to pH 7.4 and seeded into the channels by centrifugation. For each cord mold, 2 million HUVECs and 40 000 CH310t1/2s were used. Excess collagen was aspirated from the molds, leaving the cell/collagen mixture only in channels. The molds were then incubated for 8 min at 37 °C to allow collagen polymerization. Then, a drop of EGM-2 media (Lonza) was carefully introduced on top of the cords and the constructs were returned to incubation for 3 h. Following cord contraction, the molds were inverted onto a polydimethylsiloxane (PDMS) gasket containing 150 *μ*l of 10 mg/ml fibrin, which was allowed to polymerize for 5 min at 37 °C. Next, the molds were peeled away, leaving the cords embedded in the fibrin. A second layer of fibrin was added to fully encapsulate the cords. To obtain patches for implantation, punches from the larger construct were taken with a 6 mm biopsy punch.

### Maintenance and differentiation of pluripotent stem cells

Human iPSCs (WTC-11, Coriell) were maintained on Matrigel (BD Biosciences) coated plates with mTeSR-1 (Stemcell Technology). Small-molecule modulation of the Wnt pathway^25^ was used to differentiate cardiomyocytes. Briefly, iPSCs brought to a single cell suspension were seeded at a density of 42 000 cells/cm^2^ in Matrigel coated plates with mTesR containing 10 *μ*M Y-27632 (Stemcell Technology). After 24 h, media was changed to mTesR with 1 *μ*M Chiron 99021. 24 h later, media was changed to Roswell Park Memorial Institute (RPMI) 1640 media supplemented with B27 (minus insulin) containing 5 *μ*M Chiron 99021. After 48 h, cells were rinsed with PBS and media was changed to RPMI 1640 supplemented with B27 (minus insulin) containing 2 *μ*M Wnt-C59. After an additional 48 h, media was replaced with RPMI 1640 supplemented with B27 (minus insulin) without additional small molecules. 48 h later, the media was changed to the final “cardiomyocyte media” (RPMI supplemented with B27 containing insulin). From this day forward, cardiomyocyte media was replaced every 48 h. Beating clusters were observed starting on approximately day 9 of differentiation. On day 14, cells were replated on Matrigel-coated plates. Lactate selection with DMEM (no glucose) supplemented with 4 mM sodium lactate was performed between days 18 and 22.^26^ Cardiomyocytes were used at D28 post differentiation.

### Supra-epicardial implantation of patches on athymic rat hearts

All animal procedures were approved by the University of Washington Institutional Animal Care and Use Committee (IACUC protocol #4388–02). Male athymic nude Sprague-Dawley rats (Envigo, 150–220 g) underwent thoracotomy to implant 2, 6 mm patches on the supra-epicardial surface of the heart. First, animals were induced with 5% inhaled isoflurane, orotracheally intubated, and maintained on 2% isoflurane. A left lateral thoracotomy was performed, and the chest was held open with retractors to expose the heart. After removing the pericardium, patches were affixed to the supra-epicardial surface using 8–0 suture. The chest was closed, and animals recovered until they were able to move about freely. Analgesia was provided during the first two days with a subcutaneous injection (1 mg/kg) of slow releasing buprenorphine. For patches that contained cardiomyocytes, daily subcutaneous injections of Cyclosporine A (5 mg/kg) were administered for 7 days, beginning on the day prior to tissue implantation.

### Tissue harvesting, processing, and 2D histology

Animals were sacrificed at the appropriate timepoints via intra-peritoneal injection of pentobarbital/phenytoin solution (Beuthanasia; 1.5 ml injection), and the heart was removed. Explanted hearts were processed for histology by fixing in 4% (vol/vol) paraformaldehyde (PFA) for 48 h at 4 °C, progressively dehydrating the tissue with ethanol, and embedding in paraffin. Blocks were sectioned using a microtome (5 *μ*m) and collected on charged slides. Overall tissue morphology was visualized with hematoxylin and eosin (H&E). The collagen structure was identified by staining with Picrosirius Red (Direct Red 80, Sigma) using Fast Green (Sigma) as a counterstain. For immunostaining, heat mediated antigen retrieval in pH 6.0 sodium citrate buffer was performed, and sections were blocked with normal goat serum (5%). Overnight incubation of primary antibodies was performed at 4° using antibodies against human CD31 (1:20, Dako), hemoglobin A (1:100, Abcam), or beta-myosin heavy chain (hybridoma supernatant, ATCC #CRL-2046, full strength). For brightfield peroxidase-based visualization of huCD31 and CD68 (1:100, Abcam), we used either an anti-mouse or anti-rabbit specific Avidin/Biotin complex (ABC) detection kit (AbCam). For fluorescence visualization, hemoglobin A was detected with 1:500 IgG (H + L) Highly Cross-Adsorbed Donkey anti-Rabbit secondary Alexa Fluor® 488 (Invitrogen) and β-MHC or huCD31 was detected with 1:500 Novex Goat anti-Mouse IgG1 Secondary Antibody, Alexa Fluor 555 conjugate. Fluorescence images were acquired on a Nikon Eclipse Ti inverted microscope with either a Photometrics CoolSNAP HQ2 camera for widefield images or a Yokogawa W1 spinning disk confocal head and Andor iXon Life EMCCD camera for confocal images. H&E, Sirius Red, and 3′-Diaminobenzidine (DAB) stained slides were scanned using an Aperio ScanScope AT2 digital whole slide scanner.

### Retrograde perfusion, clearing, and 3D imaging

For studies on the 3D vessel structure (lectins and OMAG), n = 3 rats per group (n = 3 for lectin, n = 3 for OMAG) were implanted with cord patches containing cardiomyocytes, HUVECs, and Normal Human Dermal Fibroblasts (NHDFs) in the bulk fibrin. On day 7 post-implantation, rats were sacrificed, and hearts were fixed by retrograde perfusion. First, rats in a deep plane of anesthesia (5% isoflurane) were administered 50 U Heparin via the inferior vena cava. The heparin was allowed to circulate for 1 min, and the heart was then removed and submerged in saturated potassium chloride (KCL) solution. Next, the heart was cannulated through the aorta and flushed with vasodilation buffer (PBS containing 4 mg/l Papaverine and 1 g/l adenosine) until no blood was apparent in the effluent. To visualize vessels, 50 *μ*g/ml DyLight 649 labeled *Lycopersicon Esculentum* (Tomato) Lectin (LEL, TL) in vasodilation buffer was perfused for 10 min through the hearts. Hearts were then fixed by perfusion with 4% paraformaldehyde and either used the same day for OMAG or left in fixative overnight. The next day, hearts were rinsed with PBS and incubated with blocking solution (0.1M Tris, 1% BSA, 1% Normal Donkey Serum, 0.3% triton x-100) for 6 h at 37 °C. Then, hearts were transferred to blocking solution with 1:100 human specific CD31/PECAM-1 DyLight 550 conjugated antibody (Clone: JC/70A, Novus) for overnight incubation at 37 °C, shakings. Following staining, hearts were cleared using the Clearing Enhanced 3D Microscopy (Ce3D) protocol at 37 °C for 48 h.^17^ Cleared hearts were imaged using a Leica TCS SP8 confocal laser scanning microscope.

### Optical microangiography

Hearts that were visibly well perfused (intralipid filling small vessels) were used for OMAG imaging. The tissue was prepared for optical microangiography (OMAG) imaging following the protocol established by Qin *et al.*^19^ Briefly, the hearts were retrograde perfused with 4% PFA and then left in a bath of PFA for 1 h prior to imaging. Prior to imaging, a 10% intralipid solution was perfused through the heart at a constant pressure of 120 mm Hg for 20 min to equilibrate. After 20 min equilibration, the heart was placed onto a translating imaging stage and raised to the focal plane of the OCT imaging arm. The swept-source OCT (SS-OCT) system employed in this study has been previously reported in detail.^27^ Briefly, a 200-kHz vertical-cavity surface-emitting (VCSEL) swept laser source (SL1310V1–10048, Thorlabs Inc., Newton, NJ) with a central wavelength of 1305 nm (spectral bandwidth of 100 nm) was utilized, providing an axial resolution of ∼8 *μ*m in tissue (∼11 *μ*m in air). The power of the incident light on the sample was measured at ∼5 mW, providing an OCT sensitivity of ∼105 dB. For this study, an 18 mm effective focal length lens (LSM02, Thorlabs Inc.) was used, resulting in a lateral resolution of ∼10 um. 3D volumetric scans were acquired with a field of view (FOV) of 4 mm × 4 mm and a penetration depth of ∼1.5 mm. The beam spot was scanned using a paired X-Y galvo scanner (6210H, Cambridge Technology, Bedford, MA), forming raster sampling patterns comprising fast (x-axis) and slow (y-axis) scans. At each y-location, 400 A- scans were acquired to create a single B-frame. Eight B-frames were repeated before moving to the next y-location. Following this protocol, a single 3D volumetric scan (C-scan) was generated (a detailed analysis of typical OMAG scan sequence was previously reported by Deegan, *et al.*^28^). The repeat frames were used to generate the optical microangiography (OMAG) image based on eigen decomposition (ED) analysis.^29^ The basis of this technique allows repeat B-frames to provide a 3D volume image contrasted by particles in motion. The scan protocol was designed to contrast capillary vessels with a minimum flow rate of 0.1 mm/s. The resulting ED-based OMAG signal thus identified regions where intralipid was flowing through microvessels faster than 1 mm/s.^30^ In this case, the intralipid solution flowing in the microvessels provides 3D contrast of the vascular structure. The method resulted in a co-registered image of both static (tissue) and dynamic (intralipid) components providing information on both local tissue and the vascular structure.

The 3D structure and vascular images were captured both on healthy tissue and within the patch region. For visualization, the 3D data were compressed to maximum intensity projected en-face vascular images, with the ED-signal above a 5 dB threshold displayed and mapped to a color based on the depth.^28^ The 3D data were cropped at approximately 450 *μ*m below the tissue surface to remove any signal from beyond the vascular wall. A 10 mm FOV charge-coupled device (CCD) camera co-aligned with the OCT imaging arm captured visible light images to align the OCT images with the patch and healthy heart region.

### Quantitative tissue morphometry

For the purposes of analysis, the patch area was defined as the area spanning from the epicardial surface of the rat heart to the outer edge of the patch. Nuclear density was quantified in FIJI using the color deconvolution and automatic cell counter tools to isolate the hematoxylin and eosin stains and count hematoxylin positive nuclei within the patch area, respectively. For quantification of the CD31 and HgbA area, a biologically relevant threshold was selected and applied uniformly to all images in each set, and the percent area of the patch positive for each stain was calculated.

For analysis of the CD68+ area, images from each scanned slide were generated to represent three distinct regions of tissue. First, the patch area was defined as the region from the epicardial surface of the rat heart to the boundary of visible tissue. Second, the patch-heart interface was defined as the 300 *μ*m of heart tissue underlying the patch. Finally, a region of healthy heart was selected from heart tissue on the opposite side of the sutures. Images from these three regions were separated into stained and unstained regions using the segmentation tool Ilastik. The stained and unstained regions were converted to binary masks from which percent of stained area was calculated.^18^

For quantification of the huCD31 and LEL positive area within 3D stacks, each frame was separated into stained and unstained regions using Ilastik. The stained and unstained regions were converted to binary masks from which percent of stained area was calculated.^18^ Quantified data from 3 animals were averaged and plotted with a custom python script. 3D volumes were visualized using ChimeraX.^31^

### Nearest neighbor analysis

Images of hematoxylin and eosin (H&E) stained explanted grafts were acquired and preprocessed using the ImageJ based image-processing software FIJI. The eosinophilic red blood cell signal was separated using the built in “deconvolve” function in FIJI. The corresponding signals were thresholded and converted to binary masks. A custom python script was used to perform a distance transform of each binary mask. The output of the distance transform is an array with each pixel's intensity corresponding to its distance away from the background in the respective binary mask. The pixels corresponding to the local maxima of the distance transformed binary masks were used as indices for individual red blood cells. A graph was then constructed with nodes representing single red blood cells and edges with weights corresponding to the Euclidean distance between the two nodes (red blood cells) they connect. The corresponding adjacency matrix was used to find the minimum distance to a neighboring cell for every cell in the image.

Images acquired after immunostaining explanted grafts for human CD31 (huCD31) and staining with Hoechst were preprocessed using a morphological top-hat transformation to reduce background noise. huCD31 channels and the Hoechst channels were then isolated from the background corrected images. The individual channels were thresholded and converted to binary masks. A distance transform was performed on the Hoechst channel masks. The local maxima of the distance transformed masks were used as cell nuclei indices. These indices were then used to check for the colocalized huCD31 signal and mark huCD31-positive cells. A graph was constructed with the nodes representing single huCD31-positive cells and the edges representing the Euclidean distance between the two nodes (huCD31-positive cells) they connect. The graph's adjacency matrix was used to find the minimum distance to a neighboring cell for every cell in the image.

### Statistical analysis

Graphpad prism software was used to compare the three groups using one-way analysis of variance (ANOVA) with Tukey's honestly significant difference (HSD) as the post-hoc test. Error bars are reported as the standard error of the mean. For *in vivo* experiments, the sample size represents the number of animals in each group.

## SUPPLEMENTARY MATERIAL

See the supplementary material for H&E and Sirius red stains of cord patches implanted in the mouse abdomen.
